# Public involvement in health outcomes research: lessons learnt from the development of the recovering quality of life (ReQoL) measures

**DOI:** 10.1186/s12955-019-1123-z

**Published:** 2019-04-11

**Authors:** Andrew Grundy, Anju Devianee Keetharuth, Rosemary Barber, Jill Carlton, Janice Connell, Elizabeth Taylor Buck, Michael Barkham, Thomas Ricketts, Dan Robotham, Diana Rose, John Kay, Rob Hanlon, John Brazier

**Affiliations:** 1 School of Health Sciences, University of Nottingham, Nottingham, UK; 2 School of Health and Related Research, University of Sheffield, Sheffield, UK; 3 Department of Psychology, University of Sheffield, Sheffield, UK; 4 McPin Foundation, London, UK; 5 King’s College London, Institute of Psychiatry, London, UK; 6 Sheffield Hallam University, Sheffield, UK; 7 Derby, UK

**Keywords:** Patient reported outcome measure (PROM), Service user, Patient and public involvement (PPI), Public involvement, Co-production, Mental health, Outcome measure

## Abstract

**Background:**

To provide a model for Public involvement (PI) in instrument development and other research based on lessons learnt in the co-production of a recently developed mental health patient reported outcome measure called Recovering Quality of Life (ReQoL). While service users contributed to the project as research participants, this paper focuses on the role of expert service users as research partners, hence referred to as expert service users or PI.

**Methods:**

At every stage of the development, service users influenced the design, content and face validity of the measure, collaborating with other researchers, clinicians and stakeholders who were central to this research. Expert service users were integral to the *Scientific Group* which was the main decision-making body, and also provided advice through the *Expert Service User Group*.

**Results:**

During the theme and item generation phase (stage 1) expert service users affirmed the appropriateness of the seven domains of the Patient Reported Outcome Measure (activity, hope, belonging and relationships, self-perception, wellbeing, autonomy, and physical health). Expert service users added an extra 58 items to the pool of 180 items and commented on the results from the face and content validity testing (stage 2) of a refined pool of 88. In the item reduction and scale generation phase (stage 3), expert service users contributed to discussions concerning the ordering and clustering of the themes and items and finalised the measures. Expert service users were also involved in the implementation and dissemination of ReQoL (stage 4). Expert service users contributed to the interpretation of findings, provided inputs at every stage of the project and were key decision-makers. The challenges include additional work to make the technical materials accessible, extra time to the project timescales, including time to achieve consensus from different opinions, sometimes strongly held, and extra costs.

**Conclusion:**

This study demonstrates a successful example of how PI can be embedded in research, namely in instrument development. The rewards of doing so cannot be emphasised enough but there are challenges, albeit surmountable ones. Researchers should anticipate and address those challenges during the planning stage of the project.

## Background

The notion that people with lived experience of a health condition should be involved in designing and conducting health research has become increasingly acknowledged and valued in the United Kingdom (UK) and internationally [1–4]. Public involvement (PI) in the UK has been defined as “research carried out ‘with’ or ‘by’ members of the public rather than ‘to’, ‘about’ or ‘for’ them” [5]. Thus, PI is distinct from patients as research ‘participants’ from whom data is collected, and is focused on ‘involvement’ in the actual design and conduct of research. It is said to lead to research of a higher quality that is more acceptable, relevant, transparent and accountable [6–8]. Guidance for reporting PI in health and social care research has been developed to allow researchers to learn from best practice in different health specialties [9].

The growth of PI in health research has been uneven and, somewhat surprisingly, is often absent from the development of Patient Reported Outcome Measures (PROMs) [10]. These instruments focus on how a person interprets, perceives and feels about aspects of their health status and treatment, and are increasingly being used in clinical practice [10, 11]. The phrase ‘patient-reported’ indicates that an individual has self-completed the measure, but does not imply that the development of the PROM has been shaped by patients. In this paper, the phrase ‘service user’ will be used instead of ‘patient’, as is conventional in the field of mental health in the UK; the term ‘expert service user’ will be used to refer to PI inputs from mental health service user research partners in this particular programme of research. Despite growing recognition of the value of experiential knowledge that service users bring to health research, a recent review found that only 6.7% of PROMs had input from service users at every stage of PROM development [10]. Most of the papers (58.5%) described some involvement in PROM development, mainly with item generation, and the authors of the review suggest that some researchers may have omitted to report involvement altogether.

There is limited agreement between clinicians and service users on outcome priorities [12, 13]. When service users were consulted about the relevance and acceptability of commonly used outcome measures in mental health assessment, many were rated low as they did not reflect service users’ own concerns [14]. This has led to suggestions that outcome measures should not only embody the values and priorities of service users, but that service users themselves should be involved as key decision-makers throughout the PROM development process [10, 13, 15, 16]. In this way, the questionnaires are likely to be more relevant, comprehensive and understandable to service users, resulting in enhanced reliability and validity of the measures [10, 13]. There are limited models about how to achieve greater involvement of service users in the development of PROMs, and even fewer reports regarding the impact of service user involvement on the development of such measures [10, 17].

Recovering Quality of Life (ReQoL) is a new instrument which measures mental health service users’ own perspectives of ‘recovery’ and ‘quality of life’ [18]. It was developed from the outcomes that service users identified as being central to them, as well as from the literature [19–21]. The stages of measurement development include the identification of themes and items (Stage 1), the face and content validity with service users (Stage 2), and the psychometric testing by collecting data on the draft questionnaires (Stage 3) before finalising the measures. ReQoL is available in both a short version for clinical assessment (comprising 10 items, ReQoL-10), as well as a longer version (comprising 20 items, ReQoL-20). Both measures are suitable for self-completion and for use across a wide spectrum of mental health conditions (both psychotic and non-psychotic) and for different levels of severity, for individuals aged 16 or over. The intention was to deliver a rigorous service user-centred and service user-valued PROM with high face and content validity. In most PROM developments, patients are solely research participants providing data that are used in the process. Ethical approval to use data from patients in research is sought through the relevant authorities. This paper focuses on the involvement of expert service users as research partners with other service users as participants in the study. The aims of the paper are to provide an example of PI being deeply embedded in the development of a mental health PROM and to critically assess the contribution of expert service user involvement.

## Methods

### The role of service users in the governance of ReQoL

**Fig. 1:**
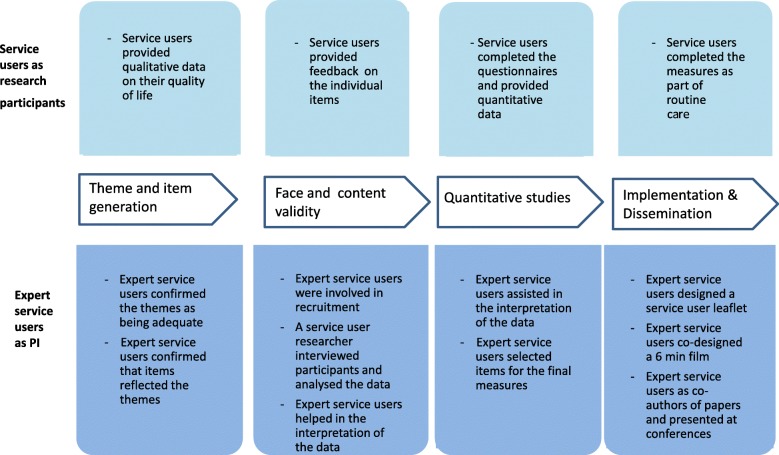
Distinct roles of service users as research participants and as PI in the development of the ReQoL

ReQoL was developed by a *core team* of seven academics and a *scientific group* (which included the core team) comprising seven expert service users, five clinicians, five academics and two clinical academics; these were the main decision-making bodies. They were supported by four advisory groups who provided opinions and recommendations at different stages of the research: (1) The *expert service user group* included two expert service users from the scientific group, plus other five other expert service users. All were purposively chosen in a number of ways. First, the research team approached people in their existing networks; second, expert service users recommended other service users to the research team and third, one expert user responded to a request circulated through a mental health network. Some had an academic background and were familiar with PROM development, others had varying experiences of research. (2) The *psychometrics group* comprised six ‘psychometricians’ who are experts in the science of measurement and the development of outcome measures. (3) The *stakeholder group* included 32 policy-makers and clinicians, while (4) the *advisory group* consisted of 33 national and international academics. Figure 1 summarises the involvement of service users in the three development stages and the implementation and dissemination of ReQoL. The top and bottom parts of the figure outline the role of service users as research participants and PI respectively. Full details of the development of ReQoL are available elsewhere [18, 22, 23].

#### Stage 1. Theme and item generation

The aims of PI in this first stage of PROM development were to validate the over-arching themes of the measure and to co-produce a pool of candidate items to be tested in the next phase. Seven broad health themes were identified by the *core team* as important to service users regarding their quality of life, these were: *activity*, *hope, belonging and relationships, self-perception, wellbeing, autonomy* and *physical health*. These potential domains were presented to the first meeting of the *scientific group* to ascertain whether they (including the expert service users) believed that the domains were appropriate*.* The *core team* then began to develop positive and negative sub-themes for each domain, and generate items that might enhance or deplete quality of life. These items were improved or removed using criteria proposed by Streiner and Norman [24]: too complex; ambiguous; double-barrelled; jargon; value-laden; negatively worded; or too lengthy.

In all the stages, there were at least 5 expert service users for the meeting to proceed but at times there have been as many as seven. The first meeting of the *expert service user group* considered the pool comprising 122 items. Details of how these were generated are discussed elsewhere [18, 22, 23]. They were clustered together by domain and written on post-it notes displayed on flipchart paper on the walls of the meeting room. During the morning, each member walked around the room and allocated ‘votes’ for each domain by placing a coloured sticker on the post-it note next to their preferred items. They modified existing items and also wrote new ones to reflect anything they thought was not quite right or missing. They applied the above criteria from their perspectives, in addition to bringing their lived experience particularly concerning the emotional impact of the items. In the afternoon, the most highly rated items were discussed and the rationale for either keeping or removing them was noted. The *advisory group* and the *scientific group* met subsequently and separately to carry out a similar exercise. The co-produced pool of candidate items was then presented to study participants in the next stage of the process.

#### Stage 2. Face and content validity testing of shortlisted items

During this stage, three experienced qualitative researchers (JCo, JCa, and AG) conducted individual interviews, paired interviews and focus groups to obtain the views of service user participants. In terms of PI, one of the interviewers was an expert service user in an academic post, who shared this status with all the participants he interviewed. Participants were asked to comment on a pool of potential items to test the content validity (the extent to which the set of items covers all the components of quality of life) and the face validity (whether the items are relevant to people who use the measure). At the end of this stage, PI consisted of members of the *expert service user group* joining with the *scientific group* to discuss the results. The aim of PI was to validate the interpretations being made by others on the research team from the qualitative data and to refine the pool of items collaboratively.

#### Stage 3. Item reduction and scale generation

The aim of PI in this stage of the process was to reduce the pool of items and then to collectively agree on the final format of the measure, including the ordering of items. Psychometric testing was achieved through two quantitative studies in the form of online and postal questionnaires completed by service user participants. Following the psychometric analyses advised by the *psychometrics group*, the *expert service user group* met separately to review the eliminated items and to try to achieve consensus on the most appropriate remaining items. The *expert service user group* and the *scientific group* met together later to discuss the psychometric performance of the different items, alongside the qualitative data from the previous stage. The *expert service user group* met again prior to joining the *scientific group* to finalise the ReQoL measures. The combined *expert service user group* and *scientific group* then collaborated to select the most appropriate items for each domain and make decisions about whether or not additional items were needed.

#### Stage 4. Implementation stage

The aim of PI in this final stage was to agree dissemination priorities and to develop creative ways to disseminate findings that would be engaging and accessible to various audiences. In line with good practice, members of the *expert service user group* were involved in disseminating the results of the ReQoL project through a film, co-producing leaflets, conference presentations and co-authoring publications.

## Results

### Stage 1. Theme and item generation

The expert service users at the *scientific group* meeting affirmed the appropriateness of the seven domains of the PROM. However, they expressed some deep concerns about the concept of ‘recovery’. The main concern was that the concept of recovery was centred on self-management and a wish to ‘normalise’ service users with mental health difficulties to conform to one convention dictated by society, rather than embracing their differences. The shared definition of recovery that was endorsed was: “You could have distressing symptoms but still have a good quality of life”. There was also some strong debate around the fact that while recovery was important, mental health services were often not funded to address the wider aspects of service users’ lives (e.g. belonging and relationships) but very much focussed on the reduction of symptoms.

At the first meeting of the *expert service user group* the 122 items were explored in-depth systematically and additional items were suggested by the group increasing the number of items to 180. Among these 58 additional items, nine were completely new items about missing sub-themes (Table 1); 22 had been dropped at much earlier stages along the selection process; and 27 items were dropped from the previous pool. As a result of the addition of items at that stage, three extra *core team* meetings had to be scheduled to carefully consider these items and comments.

**Table 1:** Items/sub-themes added by the *expert user group* in the theme and item generation stage

	Item/sub-theme added	What happened to that item?
1	Autonomy in my care	This was dropped as it was more about ‘process’ rather than ‘outcome’
2	I am satisfied in my sex life	This was dropped because it was felt that it may not be applicable to a number of service users
3	I felt stupid (self-criticism)	The concept was covered by an item on self-blame and the item ‘I felt guilty’
4	I chastised myself for my mistakes	This was covered by an item on ‘blame’
5	Dread (as opposed to anxious as being anxious can be a good thing at times)	This idea was covered by the following items ‘I felt panic / terror’ ‘I worried too much’ ‘I had difficulty stopping or controlling my worry’
6	I am bothered by the side-effects of my medication	This was dropped because it was felt that it may not be applicable to a number of service users
7	I am able to carry out day-to-day activities	This was covered by the following few items: ‘I have reasons to get out of bed in the morning’ ‘I could not get started with the simplest everyday tasks’
8	Sleeping too much	The existing item on sleep was modified to encompass all sleep-related problems
9	Consumed by anger	The item ‘I felt consumed by anger’ was added

Members of the *expert service user group* expressed some concern that the pool of items at this stage felt too symptom-based, and that certain items reflected professional priorities or phraseology (e.g. how usual is it for people to have ‘plans and goals’?). The disquiet was that the items may not reflect a broader conceptualisation of ‘quality of life’ and also of ‘recovery’ from the service user perspective. The expert service users raised questions regarding whether or not the existing measures from which some of the items were taken had themselves been co-constructed with service users. However, the research team established that there was not enough time to review this issue within the tight time frame and as long as items were thoroughly tested by service users, they should be considered even if they came from measures that were not co-constructed. Pragmatically, discussions focussed on the rationale for keeping, removing, or adding items and were detailed and intense, with different possibilities of the meanings and acceptability of words and phrases examined very carefully.

After a comparable exercise was subsequently undertaken by the *scientific group*, the items were then reduced to 101. The *core team* further reduced the number of items to 88 for use in the subsequent stage through a similar exercise guided by the Streiner and Norman criteria [24]. It was important to reduce the number of items to make the face and content validity stage practically manageable without imposing unnecessary burden to participants.

### Stage 2. Face and content validity testing of shortlisted items

In order to test face and content validity of the reduced pool of 88 items, 40 individual interviews, four paired interviews and two focus groups (*n* = 11) were carried out, obtaining the views of 59 service user participants and 19 service user participants aged 16–18. Important issues emerged from the interviews concerning the perceived irrelevance, complexity and ambiguity of certain items. Potentially distressing and judgmental items were also highlighted [22] . Mid-way through data collection the three interviewers, in conjunction with the *scientific group* and the *expert service user group*, agreed to add 12 more items as a result of feedback from the study participants.

At the *scientific group* meeting, the feedback received from the study participants on each item was discussed. In some instances, there were conflicting views between the feedback received from expert service users in the previous stage and that received from study participants. One example of disagreement concerned the item *‘I felt guilty’* which the expert service users found to be an important item. Study participants felt that at times it could be a positive thing to feel guilty in some circumstances (for instance, one is well enough to appreciate what one might have done when experiencing a serious episode), whereas in other circumstances it could be a negative experience of being too critical towards oneself. This item was discussed and it was agreed to drop it because the item not only enhances quality of life but also takes away from it. At this stage it was necessary to review the feedback from expert service users from the previous stage with the new evidence from study participants. In cases where there were disagreements (*n* = 20 items), these were highlighted by the qualitative researchers in advance of the meeting and more time was devoted to discussing such items to reach consensus on whether the item should be omitted, retained or re-worded. Therefore, as well as service users as participants, in terms of PI, an expert service user was also involved in collecting data and all the expert service users were involved in re-shaping the interview topic guide. Thus, the contribution of the expert service users was not only in terms of the contribution to the wording of items but to the underlying conceptualisation of the scale.

### Stage 3. Item reduction and scale generation

A fundamental aspiration of the expert service users involved was that completing the PROM should not leave people feeling “rubbish”, upset, or worse than they felt before completing the measure. The expert service users therefore finalised the order of the questionnaire that was to be used in the quantitative studies. The psychometric testing of the questionnaire comprised two quantitative studies, recruiting 2062 and 4266 service user participants respectively. In the former, service user participants completed a larger item-set of 61 items and in the latter, participants completed a set of 40 items. In terms of PI, it should be noted that expert service user identified by the service providers assisted in the recruitment of participants through their networks. Furthermore, following the psychometric analysis of the first study, the *expert service user group* appraised those items that had been eliminated and attempted to achieve consensus on the most appropriate remaining items. Discussions focussed on the ordering and clustering of the themes and items (e.g. should positive and negative items be separated or mixed?), and different options for items concerning physical health.

During the final stage of development after the second quantitative study, the *expert service user group* met separately before joining the *scientific group* later the same day to examine all the data and to finalise the short form (ReQoL-10) and the longer version (ReQoL-20). The combined group considered which items were most appropriate for each domain, and agreed that no additional items were needed. The expert service users contributed to the final item selection, and while this group was happy with the short ReQoL measure to contain 10 items, clinicians were of the view that six items would be sufficient. This was debated and the group agreed that 10 items offered better psychometric properties than six items. Because of the simplicity of the items of the ReQoL, the additional burden of four questions was minimal. It was also decided that the physical health item should be included in both versions of the PROM. As shown in Table 2, the result of inputs from expert service users in the decision-making process meant that the items with the strongest psychometric properties were not automatically chosen for the final measure. Instead, a compromise was reached between psychometric strength and content validity.

**Table 2:** Ranking of items by psychometric properties within each theme

Theme	Description	Psychometric properties ranked^a^
Activity	I found it difficult to get started with everyday tasks	1 out of 5
	I enjoyed what I did	4 out of 5
Belonging and relationships	I felt lonely	1 out of 5
	I felt able to trust others	3 out of 5
Choice, control and autonomy	I felt unable to cope	1 out of 5
	I could do the things I wanted to do	3 out of 5
Hope	I thought my life was not worth living	2 out of 4
	I felt hopeful about my future	4 out of 4
Self-perception	I felt confident in myself	1 out of 4
Wellbeing	I felt happy	1 out of 4

^a^This subjective ranking was based on: a. the information function produced by the Item Response Theory (*IRT*) models b. whether the item fits the IRT model and c. item-level responsiveness

### Stage 4. Implementation stage

A short video describing the ReQoL project was co-developed prior to the launch of the PROM (http://www.reqol.org.uk/p/overview.html). Expert service users helped to devise an information sheet about ReQoL, and they also attended the launch event during which barriers and facilitators surrounding the use of ReQoL were discussed. Furthermore, members of the *scientific group* met to discuss the possibilities of translating the PROM into different languages. Finally, expert service users are co-authors of published papers (including this one) and conference presentations arising from developing ReQoL.

## Discussion

### Principal findings

This paper gives an account of one of the few examples in the literature of PI at every stage of a PROM development: recruitment to studies, collecting data, interpretation and dissemination of the findings. The service user voice was heard not only from the data sources (items from existing outcome measures; qualitative interviews; face and content validity testing; and psychometric testing), but also by expert service users being actively involved in decision-making regarding the domains and the items of the PROM through their membership in the *scientific group* and the *expert service user group*. Crucially, expert service users were key collaborators in the design of the PROM. The following section presents the value added and key issues of embedding PI in the project.

### Assessing the impact of PI

#### Validating interpretations

The importance of PI in the actual design and development of a PROM cannot be overestimated. An outcome measure that does not address the priorities, concerns, concepts and values of service users in language that is understandable and acceptable, is of little worth and likely to be misleading [25, 26]. It was imperative, for example, that from the outset expert service users in the ReQoL team validated the domains of quality of life. Furthermore, having expert service users involved in the different stages of ReQoL development meant that the data and opinions gathered from service user participants were scrutinised and interpreted by expert service users. At all stages, the expert service users commented on the comprehension of the language, conceptual difficulties, suitability and acceptability of the items. They suggested eliminating some items, re-phrasing others, and proposed new items.

The possibility of missing items of significance has been raised [10]. It was therefore essential that the expert service users had the opportunity to advise on the developing pool of items, and that new potential items for each domain could be introduced. Concerns were voiced at the first *expert service user group* that the pool of items did not appear to reflect a broader conceptualisation of quality of life from the service user perspective. Some items appeared to be professionally driven and too symptoms-focused, which prompted questions about whether or not these items had been derived from questionnaires co-constructed with service users. Very few outcome measures are wholly service user-driven. Rose et al. [27] described the benefits of a wholly service user driven approach, which included close attention to the appropriateness of language, inclusion of negative issues, and a lessening of the power relationship between interviewer and interviewee.

#### Identifying jargon

One of Streiner and Norman’s [24] criteria related to the use of jargon. As the ‘insider language’ of a profession, the implication is that ‘outsiders’ are needed to ensure that all jargon is correctly identified and then eliminated from the pool of items. Academic service users are not immune from becoming encultured into this ‘insider talk’, which is why it was important that expert service users from outside academia were also involved in both the *expert service user group* and the *scientific group.* These group members brought a truly ‘lay’ perspective throughout the whole process, complementing the views and opinions of the academic expert service users.

#### Different perspectives and priorities

It has been reported that service users interrogate and interpret qualitative research interview data differently from researchers with only an academic knowledge-base, and that pooling interpretations can yield a more fruitful analytic process [28]. One priority highlighted by the expert service users concerned the emotional impact of items and of the overall PROM. At the first meeting of the *expert service user group,* it was strongly advised that completing the measure should not leave people feeling distressed. During the face and content validity interviews, the expert service user interviewer was particularly keen to explore potentially distressing items with participants so that these could be clearly identified. Recognising ‘potentially distressing’ and ‘judgemental’ items [22] could only be properly carried out by service users themselves. There was also general consensus amongst the expert service users that the first and last items of the PROM in particular should not be ‘off-putting’, a concern noted by previous authors who advised that this could also affect completion rates [25]. Once again, the expert service users were in the best position to define what these terms were likely to mean to people completing the questionnaires. Bringing concerns about the possible emotional impact of the PROM throughout the development process also served to increase the face and content validity of ReQoL.

#### Managing disagreements

Given the differing perspectives and priorities of individuals in the decision-making groups there was considerable room for disagreement. Disagreements were experienced at every phase of the process; expert service users disagreed with one another, and sometimes expert service users disagreed with academics or clinicians, and vice versa. There were conceptual disagreements, with some expert service users rejecting normative notions of ‘recovery’ that some academics seemed to accept without question, and different conceptualisations of ‘quality of life’. There were also differences of opinion about the phrasing of items, and also the ordering of items. However, achieving consensus was essential throughout the development of ReQoL. Where there was strong disagreement, items would progress to the next stage for further testing where possible. At the final stage, consensus was achieved after taking all views equally into consideration.

We think it is first important to acknowledge that disagreements will occur in the co-production of research by virtue of different perspectives. In terms of managing them, researchers should be prepared to take the time to fully listen to the expert service users’ point of view and explain theirs. A set of shared goals about what makes a good PROM, have to be agreed at the beginning of the collaboration. Any disagreement can then be related back to these core points. When these core points are not affected by the disagreement, then it is recommended that both parties agree to disagree. Successful management of disagreements relies on mutual respect, good interpersonal skills and common sense.

#### Preparation for the meetings

Prior to each meeting of the *expert service user group*, a member of the research team (AK) produced written information and a screencast, outlining the current findings and providing details of the tasks to be undertaken at the next meeting. This provided helpful orientation around potentially difficult topics. This enabled the expert service users to be sufficiently informed so that they could bring their experiential knowledge to the decision-making process.

In addition to being present at the scientific group meetings, it was important that the *expert service user group* had the opportunity to meet independently of the *scientific group* throughout the process, to ensure that group members felt free to voice their views and concerns. This attempt to address power asymmetries enabled the *expert service user group* to reach a consensus around the key messages that they wanted to bring to the *scientific group,* and ensured that they did not feel intimidated during the larger group discussions where other experts were present.

#### Time and costs

In line with best practice [2], service users in the *expert service user group* and the *scientific group* had their travel expenses reimbursed and they were paid for their time for attending the meetings and preparing for them. Including expert service users in the development process of the ReQoL added considerable time (See Table 3 for an approximation of additional times taken).

**Table 3:** Summary of key contributions of expert service users at different stages, challenges and extra resource implications

Stages	Key contributions	Challenges	Resource implications
1. Theme and item generation	Ensured that themes were relevant and that no themes were missing Ensured that items generated were meaningful		Time for PI to prepare for (1 day) and attend 2 face-to-face meetings (2 days^a^).
2. Face and content validity	Co-produced the topic guide A service user researcher was involved in the interviewing and data analysis The data analysis was reviewed by expert service users to help to reduce the number of items, ensuring the face and content validity of the measures At the end of this stage, we were confident that no important item was missing and that items were appropriate	At the meeting to reduce the number of items (by about 40), expert service users added 58 extra items Disagreement on certain items	Time for PI to prepare for (half day) and attend 2 face-to-face meetings (2 days ^a^) Time for researcher to prepare materials for Expert Service Users Group Meeting (2 days), attend the meeting (1.5 days), write up the feedback of the meeting (2 days) Added 3 more weeks to the timeline to schedule 2 more core team meetings to reduce the number of items
3. Quantitative studies	Helped in making the final item selection Ensured that the measures had face validity	Conveying psychometrics results to the expert service user group Disagreements on certain items Trade-off between psychometric properties and face validity	3 days for PI to prepare for meetings and 2 days to attend meetings
4. Implementation and dissemination	Contributed to making the research more accessible to others	Required support to work on papers and presentations	4 days of PI work to prepare and be involved in the filming.

^a^Depending on the location of the meetings, for some expert service users attending a face-to-face took a whole day for others it might be half a day

Whilst the advantages of involving the expert service users in developing ReQoL were clearly evident, and there are examples of successful expert service user led PROM development [13], it is not known if the process of involvement described here would be suitable for developing PROMs in all different specialties. This critical assessment is based on the shared reflections of the authors. A formal evaluation of the impact of service user involvement on the PROM development, on expert service users and on the other researchers would have provided a more detailed and authoritative appraisal. The expert service users were purposively invited for their substantial expert knowledge and experience, and the need to ensure diversity including differing perspectives was not addressed; this was an omission. Despite these limitations, we believe that embedding service user inputs, their priorities, values, views and perspectives at every stage of the development of ReQoL led to a PROM that is more acceptable and meaningful to those who complete the measure.

## Conclusions

While the reflections on PI presented above are applicable to PI in research in general, the main contribution of this paper is to provide an example of how PI was successfully embedded in every stage of the PROM development. On the basis of the findings presented here, we recommend that researchers involved in future PROM development consider (Table 4): how to involve service users in every phase of the development process; extensive service user involvement is adequately planned and budgeted for; outcome measures from which items are taken are first checked that they have been co-constructed by service users and if not whether the items are acceptable to service users; the fact that expert service users are diverse; expert service users are able to reflect the views of other service users; expert service users are involved in recruitment to studies and employed in data collection and analysis; issues of power asymmetry are addressed; expert service users have an opportunity to meet independently to voice their views and concerns, and that they are appropriately briefed; research teams are prepared to resolve disagreements by having some clear guidelines from the beginning about how to reach a resolution; researchers are prepared to devote time and effort to make technical materials accessible to expert users; and the impact of expert service user involvement throughout the PROM developmental process is evaluated.

**Table 4:** Key recommendations for PROM developers

Recommendations for PROM developers: • Consider how to involve service users in every phase of the development process • Extensive service user involvement needs to be adequately planned and budgeted for • Outcome measures from which items are taken are first checked that they have been co-constructed by service users and if not whether the items are acceptable to service users • Include expert service users who are diverse • Include expert service users who are able to reflect the views of other service users • Involve expert service users in recruitment to studies, in data collection and analysis • Provide service users with an opportunity to meet independently to voice their views and concerns and ensure that they are appropriately briefed • Research teams should prepared to resolve disagreements by having some clear guidelines from the beginning about how to reach a resolution • Researchers should be prepared to devote time and effort to make technical materials accessible to expert users • How the impact of service user involvement throughout the PROM developmental process should be considered.	

The embedding of expert users in co-producing the ReQoL ensured that the measures were more meaningful to service users, thus increasing the face and content validity of the measure. Having service users as research partners making shared decisions throughout the research process was critical in producing a service user-centred and service user-valued PROM.

## Abbreviations

IRT: Item response theory
PI: Public involvement
PROM: Patient Reported Outcome Measure
ReQoL: Recovering Quality of Life
